# The association between obstructive sleep apnoea and diabetic peripheral neuropathy in subjects with type 2 diabetes

**DOI:** 10.3389/fendo.2025.1643826

**Published:** 2025-08-29

**Authors:** Na Lu, Gang Cheng, Yu Qian, Di An, Fuzai Yin, Yajing Hou, Xiaoli Liu, Qiang Lu, Chunming Ma, Rui Wang

**Affiliations:** ^1^ Department of Endocrinology, The First Hospital of Qinhuangdao, Qinhuangdao, Hebei, China; ^2^ Department of Endocrinology, Dongfang Hospital, Beijing University of Chinese Medicine, Qinhuangdao Hospital (Qinhuangdao Hospital of Traditional Chinese Medicine), Qinhuangdao, China; ^3^ Department of Respiratory, Qingdao Women and Children’s Hospital, Qingdao, Shandong, China; ^4^ Department of Pharmacy, The First Hospital of Qinhuangdao, Qinhuangdao, China; ^5^ Department of Ophthalmology, The First Hospital of Qinhuangdao, Qinhuangdao, China

**Keywords:** obstructive sleep apnea, diabetic peripheral neuropathy, diabetes mellitus, type 2, apnea-hypopnea index, corneal nerve fiber

## Abstract

**Objective:**

This study aimed to examine the association between obstructive sleep apnoea (OSA) and diabetic peripheral neuropathy (DPN) in subjects with type 2 diabetes mellitus (T2DM).

**Methods:**

A cross-sectional study was conducted involving 228 T2DM subjects at The First Hospital of Qinhuangdao. OSA was assessed using polysomnography. DPN was diagnosed based on clinical signs, symptoms and electromyography findings. Small fibre neuropathy was additionally assessed through corneal confocal microscopy. Among these T2DM subjects, 124 (54.4%) had DPN. The prevalence of OSA was 67.5% (mild OSA 30.7%, moderate-to-severe OSA 36.8%). DPN prevalence rates were 40.5%, 52.9% and 67.9% in subjects without OSA, with mild OSA, and with moderate-to-severe OSA respectively. Multiple logistic regression analysis revealed that moderate-to-severe OSA was independently associated with DPN in T2DM subjects (AOR=2.176, 95%CI:1.050-4.511, p=0.037). Multiple linear regression analysis demonstrated that apnea hypopnea index (AHI) was independently associated with corneal nerve fiber length (CNFL)(coefficient=-0.032, p=0.049, R^2^ = 0.029) and CNFT (coefficient=0.023, p<0.001, R^2^ = 0.171) in T2DM subjects.

**Conclusion:**

T2DM subjects with OSA demonstrate significantly higher odds of DPN. Furthermore, OSA shows a significant correlation with small fibre damage in T2DM subjects.

## Introduction

Diabetes mellitus is a global metabolic disorder characterised by chronic hyperglycaemia resulting from impaired insulin secretion, insulin action, or both. The International Diabetes Federation (IDF) Diabetes Atlas (11th edition) reports that approximately 589 million adults (20–79 years) currently live with diabetes worldwide - representing 1 in 9 adults. Projections indicate this number will rise to 853 million by 2050 (https://diabetesatlas.org/). China has experienced a dramatic increase in diabetes prevalence in recent decades, with national survey data demonstrating a diabetes prevalence rate of 12.4% in 2018 (1). Type 2 diabetes mellitus (T2DM) accounts for over 90% of diabetes cases in China (2). T2DM affects multiple major organ systems, including the cardiovascular system, nervous system, eyes, and kidneys. Diabetic peripheral neuropathy (DPN), a frequent complication of T2DM, affects approximately 67.6% of T2DM subjects in China (3, 4). Clinical manifestations of DPN include numbness, reduced pain sensation, burning sensations, and sharp pains. Without proper management, DPN may progress to serious foot complications including ulcers, and musculoskeletal damage (5). From a socioeconomic perspective, DPN contributes significantly to both direct and indirect healthcare expenditures (3).

Hyperglycaemia and diabetes duration significantly contribute to DPN development in T2DM subjects. Clinical trial evidence demonstrates that intensive glycaemic control prevents neuropathy in type 1 diabetes subjects. However, for T2DM subjects, intensive therapy shows less robust effects on clinical neuropathy outcomes, with no statistically significant benefit (6). Multiple studies indicate that beyond hyperglycaemia, additional risk factors including advanced age, visceral obesity and dyslipidaemia likely contribute to DPN pathogenesis in T2DM subjects (7, 8). Consequently, investigating these non-glycaemic risk factors is clinically crucial for developing more effective DPN prevention and management strategies in T2DM subjects.

Obstructive sleep apnoea (OSA) represents a prevalent comorbidity of T2DM, capable of affecting multiple systems and inducing multi-organ damage. T2DM subjects exhibit approximately 50% greater risk of developing OSA compared to non-diabetic individuals (9). Among hospitalised T2DM subjects, OSA prevalence exceeds 60% (10). Early meta-analyses indicated a significant correlation between OSA and neuropathy in type 1 diabetes subjects, though not in T2DM subjects (11). However, variability in OSA and diabetic neuropathy definitions across included studies introduced substantial heterogeneity (12–15). Tahrani et al. demonstrated a novel independent association between OSA and DPN in T2DM subjects (15). Dhiman et al. similarly reported higher DPN prevalence among T2DM subjects with OSA versus those without (47% vs 26.1%, p=0.02) (16). Recent large-scale retrospective cohort studies have further substantiated that T2DM subjects with OSA diagnosis face elevated DPN risk compared to their non-OSA counterparts (17, 18). These studies primarily involved European and American populations. Three Chinese cross-sectional studies investigating the relationship between the apnoea-hypopnoea index (AHI) - a key OSA severity metric - and DPN yielded inconsistent results (19–21). Consequently, more extensive research on Chinese populations is warranted to elucidate the OSA-DPN association in T2DM subjects.

Electromyography assessment, a method commonly used to evaluate DPN in previous studies, primarily detects large fibre neuropathy. Diabetes mellitus also affects small fibres, with small fibre damage progressing more rapidly in T2DM subjects (22). Small fibre impairment typically precedes large fibre damage and contributes to clinically significant outcomes including painful diabetic neuropathy and foot ulceration (23). However, small fibre function has rarely been assessed in previous research (24).

This study aimed to investigate the association between OSA and DPN in subjects with T2DM This study aimed to investigate the association.

## Methods

### Subjects

We conducted a cross-sectional study involving inpatients with T2DM from The First Hospital of Qinhuangdao between September 2020 and December 2023. T2DM diagnosis was established according to the Chinese guidelines for T2DM management (25). The inclusion criteria included: 1) all participants had confirmed T2DM, and 2) both male and female subjects aged over 18 years. Exclusion criteria comprised: 1) type 1 diabetes, other specific diabetes types, or unclassified diabetes; 2) current diabetic ketoacidosis or hyperosmolar hyperglycaemic state; 3) pregnancy; 4) active infection; 5) OSA treatment with continuous positive airway pressure, upper airway surgery, or oral appliances; and 6) neuropathy from other causes (including neurotoxic drugs, vitamin B12 deficiency, or chronic inflammatory demyelinating polyradiculoneuropathy). The Ethics Committee of The First Hospital of Qinhuangdao approved the study protocol (Approval number: 2023KZ020), and all subjects provided written informed consent prior to enrolment.

### Data collection

Data were extracted from the Hospital Information System including: 1) clinical data: age, sex, diabetes duration, and diagnoses of diabetes and hypertension; 2) anthropometric measurements: height (cm), weight (kg), waist circumference (WC, cm), and blood pressure. Blood pressure measurements were obtained using an Omron HEM-7122 automated electronic sphygmomanometer, with three measurements taken while subjects were seated after 10 minutes of rest; the average of these measurements was used for analysis. Hypertension was defined as systolic blood pressure (SBP) ≥140 mmHg and/or diastolic blood pressure (DBP) ≥90 mmHg, or subjects with a previous history of hypertension who are currently taking antihypertensive drugs (26). Body mass index (BMI, kg/m²) was calculated as weight in kilograms divided by height in metres squared. Abdominal obesity was defined as WC ≥90 cm in males and ≥85 cm in females; 3) biochemical indicators: fasting plasma glucose (FPG), glycated haemoglobin (HbA1c), triglycerides (TG), and high-density lipoprotein cholesterol (HDL-C). Dyslipidaemia was defined as TG ≥1.7 mmol/L or HDL-C <1.04 mmol/L (25).

### Obstructive sleep apnea

All study subjects underwent overnight polysomnography using the Alice Night One home sleep breathing monitor (Philips Respironics Inc., Murrysville, Pennsylvania, United States) during hospitalisation. The monitoring assessed nasal airflow, snoring, respiratory effort, pulse oximetry (SpO_2_), pulse rate, and body position. An experienced investigator blinded to participant identity conducted the measurements for one night during hospitalisation. Apnoea was defined as a ≥90% reduction in airflow lasting ≥10 seconds. Hypopnoea was defined as either a ≥30% reduction in airflow with ≥4% oxygen desaturation lasting ≥10 seconds, or a ≥50% reduction in airflow with either ≥3% oxygen desaturation or subsequent arousal lasting ≥10 seconds. The apnoea-hypopnoea index (AHI) was calculated as the number of apnoea and hypopnoea events per hour of sleep, with obstructive sleep apnoea (OSA) defined as AHI ≥5 events/hour (mild: 5-<15 events/hour; moderate-to-severe: ≥15 events/hour) (27).

### Diabetic peripheral neuropathy

The diagnosis of DPN was established based on clinical signs, symptoms, and electromyography findings. Subjects were evaluated for numbness and pain symptoms, along with neurological examinations comprising ankle reflexes, vibration perception (using a 128-Hz tuning fork), light touch sensation (assessed with a 10-g monofilament), thermal discrimination (cold/hot), and pinprick sensation during hospital admission.

The posterior tibial nerve, common peroneal nerve, sural nerve, and superficial peroneal nerve in both lower limbs were examined using a Nicolet Viking IV electromyography system (American) to assess latency, amplitude, and conduction velocity. All measurements were conducted by a qualified medical professional.

DPN was diagnosed when participants met any of the following criteria: (1) presence of ≥1 clinical sign and ≥1 symptom, (2) ≥2 characteristic symptoms, or (3) confirmed abnormal nerve conduction (2). Further details regarding the DPN diagnostic procedure are provided in the **Supplementary Material**.

### Corneal confocal microscopy

All subjects underwent additional examination using laser-scanning *in vivo* confocal microscopy (IVCM) with a 670 nm wavelength laser (HRT3; Heidelberg, Germany). The examinations were conducted in morning sessions. The confocal system provided real-time 800× magnification of scanned structures, with each image demonstrating 1 µm lateral resolution within a 400 µm × 400 µm field of view. Scanning focused on the epithelial subbasal nerve plexus in the corneal pupillary region at depths ranging from 30 to 90 µm. Approximately 50–100 images were acquired per subject and stored securely. Six high-quality subepithelial corneal nerve fiber images were successfully captured for each eye, meeting predefined quality criteria: adequate grayscale contrast between nerve fibers and background, clearly defined nerve fiber boundaries (without blurring or halos), and minimal artefacts (such as tear film reflections, bubbles, or epithelial debris) covering less than 5% of the imaging field. A single experienced ophthalmic technician performed all procedures. Image analysis was conducted using ACCMetrics software (University of Manchester, UK). The final analysis involved averaging the values obtained from the six images. The following parameters were quantified: (1) Corneal nerve fibre length (CNFL): defined as the total length of all nerve fibres and their branches (expressed in mm/mm² of corneal tissue); (2) Corneal nerve fibre density (CNFD): calculated as the number of main nerve trunks per mm² of corneal tissue; (3) Corneal nerve branch density (CNBD): enumerated as the branches originating from main nerve trunks per mm² of corneal tissue; (4) Corneal nerve fibre tortuosity (CNFT): graded on a 0–4 scale where 0 represents predominantly straight fibres; 1 denotes slightly curved fibres; 2 indicates moderately curved fibres with frequent minor directional changes; 3 corresponds to markedly curved fibres with substantial directional alterations; and 4 designates severely tortuous fibres exhibiting abrupt and frequent directional changes (28).

### Statistical analyses

Analyses were performed using STATA version 16.0 (STATA Corporation, TX, USA). Categorical data were compared using the χ² test. Quantitative data were expressed as mean with standard deviation. Continuous variables with skewed distributions, such as duration of diabetes and TG, were ln-transformed to reduce skewness and are expressed as medians with interquartile ranges. Comparisons were conducted between groups using analysis of variance (ANOVA), with Bonferroni *post-hoc* correction. Multiple logistic regression was used to model the relationship between OSA and DPN. Pearson’s correlation coefficient was used to measure the strength of association between variables. Multiple linear regression analyses examined relationships between corneal confocal microscopy parameters and other variables. Variance inflation factor (VIF) values greater than 10 were considered to indicate significant multicollinearity. p<0.05 was considered statistically significant.

## Results

This study included 228 T2DM subjects (133 males, 95 females) with a mean age of 54.2 ± 12.1 years and mean diabetes duration of 7.2 ± 6.8 years. Among these subjects, 124 (54.4%) had DPN. OSA prevalence was 67.5% (mild OSA 30.7%, moderate-to-severe OSA 36.8%). The DPN frequency was significantly higher in subjects with OSA compared to those without (61.0% vs 40.5%; χ²=8.466, p=0.004).

The patient characteristics stratified by OSA status are presented in **Table 1**. Compared to non-OSA subjects, those with moderate-to-severe OSA demonstrated significantly higher age levels and male predominance (p<0.05). Both mild and moderate-to-severe OSA groups exhibited increased smoking frequencies relative to the non-OSA group (p<0.05). Waist circumference measurements and abdominal obesity prevalence showed a positive correlation with OSA severity (p<0.05). Hypertension occurrence was significantly elevated in the moderate-to-severe OSA cohort compared to OSA-negative subjects (p<0.05). No intergroup differences were observed in TG, HDL-C, HbA1c or FPG levels (p>0.05).

**Table 1:** Characteristics of subjects with T2DM by the status of OSA.

Variable	Without OSA (n=74)	Mild OSA (n=70)	Moderate to Severe OSA (n=84)	*F orχ* ^2^	p
Age (years) mean (SD)	50.8 (12.2)	54.2 (11.5)	57.1 (11.8)a	5.56	0.004
Gender [Male (%)]	36 (48.7)	39 (55.7)	58 (69.1)a	7.021	0.030
Smoking [Number (%)]	14 (18.9)	27 (38.6)a	29 (34.5)a	7.443	0.024
Drinking [Number (%)]	14 (18.9)	22 (31.4)	26 (31.0)	3.793	0.150
Duration of diabetes (years) median (IQR)	5.0 (2.0~7.0)	6.0 (2.0~10.0)	6.0 (2.0~14.0)	2.22	0.112
BMI (kg/m^2^) mean (SD)	26.5 (4.0)	27.4 (3.5)	28.5 (3.4)a	6.09	0.003
WC (cm) mean (SD)	89.4 (9.8)	93.9 (8.7)a	99.2 (10.1)ab	20.7	<0.001
Abdominal obesity [Number (%)]	37 (50.0)	49 (70.0)a	72 (85.7)ab	23.609	<0.001
SBP (mmHg) mean (SD)	136.8 (19.8)	136.1 (20.4)	140.3 (17.7)	1.12	0.329
DBP (mmHg) mean (SD)	87.8 (10.9)	87.3 (12.8)	89.3 (12.4)	0.63	0.535
Hypertension [Number (%)]	30 (40.5)	35 (50.0)	51 (60.7)a	6.438	0.040
TG (mmol/L) median (IQR)	1.59 (1.22~2.74)	2.00 (1.56~2.59)	1.91 (1.51~2.69)	0.36	0.696
HDL-C (mmol/L) mean (SD)	0.97 (0.24)	0.98 (0.24)	0.93 (0.22)	1.26	0.287
Dyslipidaemia [Number (%)]	54 (73.0)	58 (82.9)	73 (86.9)	5.185	0.075
HbA1c (%) mean (SD)	8.87 (2.08)	9.19 (2.14)	8.54 (1.83)	1.98	0.141
FPG (mmol/L) mean (SD)	8.51 (3.04)	8.55 (3.09)	7.97 (2.56)	0.99	0.373
AHI (times/hour) mean (SD)	2.84 (1.42)	9.82 (3.18)a	29.14 (13.13)ab	219.57	<0.001

AHI, apnea hypopnea index; BMI, body mass index; DBP, diastolic blood pressure; FPG, fasting plasma glucose; HbA1c, glycosylated haemoglobin A1c; HDL-C, high-density lipoprotein cholesterol; IQR, interquartile range; OSA, obstructive sleep apnea; SBP, systolic blood pressure; SD, standard deviation; T2DM, type 2 diabetes mellitus; TG, triacylglycerol; WC, waist circumference. a compared with subjects without OSA, p<0.05, b compared with subjects with mild OSA, p<0.05.

The frequencies of vibration perception impairment were higher in subjects with moderate to severe OSA than in those without OSA (p<0.05; **Table 2**). The DPN frequencies were 40.5% in subjects without OSA, 52.9% in those with mild OSA, and 67.9% in those with moderate to severe OSA. Subjects with moderate to severe OSA were more likely to have DPN (OR=3.096, 95% CI: 1.613-5.943, p=0.001) compared to subjects without OSA. In multiple logistic regression analysis with DPN as the dependent variable, covariates were age, gender (male=1, female=2), smoking (yes=1, no=0), drinking (yes=1, no=0), diabetes duration, HbA1c, hypertension (yes=1, no=0), abdominal obesity (yes=1, no=0), dyslipidaemia (yes=1, no=0), and OSA (without=0, mild=1, moderate to severe=2). Age (AOR=1.075, 95% CI: 1.045-1.107, p<0.001), diabetes duration (AOR=1.288, 95% CI: 1.038-1.598, p=0.021), and moderate to severe OSA (AOR=2.176, 95% CI: 1.050-4.511, p=0.037) were independent risk factors for DPN in T2DM subjects (**Table 3**).

**Table 2:** The signs, symptoms, and the results of electromyography and corneal confocal microscopy in subjects with T2DM by the status of OSA.

Variable	Without OSA (n=74)	Mild OSA (n=70)	Moderate to Severe OSA (n=84)	*F orχ* ^2^	p
Signs					
Numbness [Number (%)]	17 (23.0)	18 (25.7)	30 (35.7)	3.521	0.172
Pain [Number (%)]	8 (10.8)	14 (20.0)	21 (25.0)	5.262	0.072
Symptoms					
Ankle reflexes [Number (%)]	13 (17.6)	15 (21.4)	24 (28.6)	2.815	0.245
Vibration perception [Number (%)]	19 (25.7)	28 (40.0)	41 (48.8)a	8.968	0.011
Light-touch perception [Number (%)]	7 (9.5)	11 (15.7)	15 (17.9)	2.367	0.306
Thermal discrimination [Number (%)]	9 (12.2)	10 (14.3)	17 (20.2)	2.102	0.350
Pinprick sensation [Number (%)]	7 (9.5)	9 (12.9)	11 (13.1)	0.598	0.742
Electromyography					
Abnormal nerve conduction [Number (%)]	19 (25.7)	24 (34.3)	34 (40.5)	3.865	0.145
Confocal microscopy					
CNFL (mm/mm^2^) mean (SD)	18.1 (3.3)	16.7 (3.6)a	15.5 (3.2)a	11.43	<0.001
CNFD (number/mm^2^) mean (SD)	30.2 (6.3)	28.1 (7.2)	26.8 (6.1)a	5.38	0.005
CNBD (number/mm^2^) mean (SD)	48.8 (17.0)	42.9 (18.0)	39.7 (18.3)a	5.25	0.006
CNFT (points) mean (SD)	1.66 (0.75)	2.24 (0.77)a	2.57 (0.78)ab	28.11	<0.001

CNBD, corneal nerve branch density; CNFD, corneal nerve fibre density; CNFL, corneal nerve fiber length; CNFT, corneal nerve fiber tortuosity; OSA, obstructive sleep apnea; SD, standard deviation; T2DM, type 2 diabetes mellitus. a compared with subjects without OSA, p<0.05, b compared with subjects with mild OSA, p<0.05.

**Table 3:** The relationship between OSA and DPN in subjects with T2DM.

OSA	n (%)	Model 1		Model 2	
		OR (95%CI)	p	AOR (95%CI)	p
Without OSA (n=74)	30 (40.5)	1		1	
Mild OSA (n=70)	37 (52.9)	1.644 (0.850∼3.182)	0.140	1.237 (0.589∼2.600)	0.574
Moderate to Severe OSA (n=84)	57 (67.9)	3.096 (1.613∼5.943)	0.001	2.176 (1.050∼4.511)	0.037

Model 1: Univariate logistic regression analysis with DPN as dependent variable and OSA (Without OSA=0, Mild OSA=1, Moderate to Severe OSA=2) as covariates. Model 2: Multivariate logistic regression analysis with DPN as dependent variable and age, gender (Males=1, Females=2), smoking (Yes=1, No=0), drinking (Yes=1, No=0), duration of diabetes, HbA1c, hypertension (Yes=1, No=0), abdominal obesity (Yes=1, No=0), dyslipidaemia (Yes=1, No=0), and OSA (Without OSA=0, Mild OSA=1, Moderate to Severe OSA=2) as covariates. AOR, adjusted odds ratio; CI, confidence interval; DPN, diabetic peripheral neuropathy; HbA1c, glycosylated haemoglobin A1c; OR, odds ratio; OSA, obstructive sleep apnea; T2DM, type 2 diabetes mellitus.

Subjects with moderate to severe OSA had lower CNFL, CNFD and CNBD levels than those without OSA (p<0.05). CNFL levels were lower in subjects with mild OSA than in those without OSA (p<0.05). CNFT levels were higher in subjects with mild OSA and moderate to severe OSA than in those without OSA (p<0.05). CNFT levels were higher in subjects with moderate to severe OSA than in those with mild OSA (p<0.05) (**Table 2**). AHI was negatively correlated with CNFL (r=-0.216, p=0.001), CNFD (r=-0.202, p=0.002) and CNBD (r=-0.151, p=0.023), and positively correlated with CNFT (r=0.449, p<0.001). Corneal confocal microscopy parameters were also associated with age and diabetes duration (p<0.05) (**Table 4**). In multiple linear regression analysis with corneal confocal microscopy parameters as dependent variables, and age, gender (male=1, female=2), smoking (yes=1, no=0), drinking (yes=1, no=0), diabetes duration, BMI, WC, SBP, DBP, TG, HDL-C, HbA1c, and AHI as covariates, AHI was independently related to CNFL (coefficient=-0.032, p=0.049, R^2^ = 0.029) and CNFT (coefficient=0.023, p<0.001, R^2^ = 0.171) in T2DM subjects. All VIF values were lower than 10 (**Table 5**).

**Table 4:** Simple correlations between the parameters of corneal confocal microscopy, and other variables in subjects with T2DM.

Variable	CNFL (mm/mm^2^)		CNFD (number/mm^2^)		CNBD (number/mm^2^)		CNFT (points)	
	*r*	p	*r*	p	*r*	p	*r*	p
Age (years)	-0.266	<0.001	-0.245	<0.001	-0.228	<0.001	0.393	<0.001
Gender (Males=1, Females=2)	0.082	0.216	0.079	0.233	0.062	0.351	0.014	0.834
Smoking (Yes=1, No=0)	-0.147	0.027	-0.170	0.010	-0.096	0.148	0.098	0.142
Drinking (Yes=1, No=0)	-0.107	0.107	-0.093	0.161	-0.053	0.422	0.036	0.587
Duration of diabetes (years)	-0.249	<0.001	-0.263	<0.001	-0.261	<0.001	0.233	<0.001
BMI (kg/m^2^)	-0.053	0.423	-0.024	0.719	-0.027	0.686	0.087	0.192
WC (cm)	-0.129	0.053	-0.077	0.247	-0.077	0.248	0.182	0.006
SBP (mmHg)	-0.150	0.024	-0.171	0.010	-0.059	0.378	0.168	0.011
DBP (mmHg)	0.021	0.755	-0.008	0.905	0.091	0.173	-0.035	0.599
TG (mmol/L)	0.063	0.343	0.109	0.100	0.057	0.393	-0.038	0.572
HDL-C (mmol/L)	-0.055	0.406	-0.053	0.423	-0.054	0.420	0.021	0.752
HbA1c (%)	-0.046	0.488	-0.024	0.716	-0.063	0.344	0.028	0.680
FPG (mmol/L)	-0.062	0.348	-0.035	0.598	-0.055	0.407	-0.009	0.897
AHI (times/hour)	-0.216	0.001	-0.202	0.002	-0.151	0.023	0.449	<0.001

AHI, apnea hypopnea index; BMI, body mass index; CNBD, corneal nerve branch density; CNFD, corneal nerve fibre density; CNFL, corneal nerve fiber length; CNFT, corneal nerve fiber tortuosity; DBP, diastolic blood pressure; FPG, fasting plasma glucose; HbA1c, glycosylated haemoglobin A1c; HDL-C, high-density lipoprotein cholesterol; SBP, systolic blood pressure; T2DM, type 2 diabetes mellitus; TG, triacylglycerol; WC, waist circumference.

**Table 5:** Multiple linear regression analyses for the parameters of corneal confocal microscopy (stepwise method).

Parameters	Model	Coefficients	Std. Error	*t*	p	95% CI	R^2^	VIF
CNFL (mm/mm^2^)	(Constant)	21.199	1.058	20.04	<0.001	19.114~23.284		
	Age (years)	-0.059	0.020	-2.97	0.003	-0.098~-0.020	0.051	1.23
	Duration of diabetes (years)	-0.347	0.152	-2.27	0.024	-0.647~-0.046	0.039	1.17
	AHI (times/hour)	-0.032	0.016	-1.98	0.049	-0.063~-0.001	0.029	1.08
	Smoking (Yes=1, No=0)	-1.225	0.480	-2.55	0.011	-2.171~-0.279	0.024	1.05
CNFD (number/mm^2^)	(Constant)	36.242	2.013	18.01	<0.001	32.276~40.208		
	Age (years)	-0.112	0.037	-3.00	0.003	-0.185~-0.038	0.048	1.19
	Duration of diabetes (years)	-0.811	0.289	-2.81	0.005	-1.380~-0.242	0.050	1.17
	Smoking (Yes=1, No=0)	-2.885	0.902	-3.20	0.002	-4.663~-1.108	0.035	1.02
CNBD (number/mm^2^)	(Constant)	54.445	5.763	9.45	<0.001	43.088~65.802		
	Age (years)	-0.275	0.105	-2.63	0.009	-0.481~-0.069	0.040	1.22
	Gender (Males=1, Females=2)	5.109	2.398	2.13	0.034	0.382~9.835	0.012	1.07
	Duration of diabetes (years)	-2.497	0.802	-3.11	0.002	-4.078~-0.915	0.054	1.17
CNFT (points)	(Constant)	0.645	0.219	2.94	0.004	0.213~1.077		
	Age (years)	0.022	0.004	5.43	<0.001	0.014~0.030	0.123	1.05
	AHI (times/hour)	0.023	0.003	6.68	<0.001	0.016~0.030~	0.171	1.05

Multiple linear regression analysis with the parameters of corneal confocal microscopy as dependent variable and age, gender (Males=1, Females=2), smoking (Yes=1, No=0), drinking (Yes=1, No=0), duration of diabetes, BMI, WC, SBP, DBP, TG, HDL-C, HbA1c, and AHI as covariates. AHI, apnea hypopnea index; BMI, body mass index; CI, confidence interval; CNBD, corneal nerve branch density; CNFD, corneal nerve fibre density; CNFL, corneal nerve fiber length; CNFT, corneal nerve fiber tortuosity; DBP, diastolic blood pressure; HbA1c, glycosylated haemoglobin A1c; HDL-C, high-density lipoprotein cholesterol; SBP, systolic blood pressure; TG, triacylglycerol; VIF, variance inflation factor; WC, waist circumference.

## Discussion

Our study demonstrated that DPN was a common complication among inpatients with T2DM. The prevalence of DPN increased with OSA severity, with the most pronounced effects observed in moderate to severe OSA. Within the moderate to severe OSA group, approximately two-thirds of subjects exhibited DPN. These subjects were older and had a longer duration of diabetes compared to those without OSA. To account for confounding factors, multiple logistic regression analysis was performed, confirming that moderate to severe OSA was independently associated with DPN in subjects with T2DM.

In 2012, a UK study investigated the association between OSA and DPN in subjects with T2DM. OSA was defined using the apnoea-hypopnoea index (AHI), while DPN was assessed using the Michigan Neuropathy Screening Instrument (MNSI). The study population comprised White Caucasians and South Asians. The relationship between OSA and DPN was observed regardless of ethnicity; however, ethnic differences were noted in the strength of this association. Specifically, the prevalence of DPN was significantly higher in White Caucasians with OSA (66%) than in those without (22%; p<0.001), whereas among South Asians, the difference was less pronounced (48% vs. 29%, respectively; p=0.049) (15). These findings highlight the need for further research into the relationship between OSA and DPN across different ethnic groups. Three Chinese studies have examined the OSA-DPN association. All used AHI to define OSA and nerve conduction velocity to assess DPN (19–21). Two studies showed results consistent with our findings (19, 21). However, Du et al. reported no significant AHI-DPN association (20). This discrepancy may stem from elevated glucose levels in Du et al.’s study, where mean HbA1c reached 9.64% versus approximately 8.5% in other studies. This pronounced hyperglycaemia likely dominated DPN pathogenesis, potentially masking OSA’s independent effect.

Small fiber neuropathy (SFN) is a specific type of DPN that affects small-diameter sensory and/or autonomic axons. It manifests as autonomic neuropathy and paraesthesia, characterised by pain, numbness, coldness, and burning sensations (29). Abnormal thermal (cold/hot) discrimination and impaired pinprick sensation are also common symptoms of SFN (2). In our study, pain and thermal discrimination abnormalities were more frequently observed in subjects with moderate to severe obstructive sleep apnoea (OSA), though this difference was not statistically significant.

Intraepidermal nerve fibre density (IENFD) is considered an accurate and reproducible measure of SFN and the gold standard for SFN (30). Altaf QA et al. reported that AHI was associated with lower IENFD in subjects with T2DM (24). However, IENFD requires skin punch biopsy, an invasive procedure. Corneal confocal microscopy represents a reliable and non-invasive alternative to IENFD (31). In this study, small fibre function was evaluated by corneal confocal microscopy. Corneal confocal microscopy is a useful diagnostic tool for SFN in T2DM (32). Corneal confocal microscopy correlates with peripheral nerve structure and function in T2DM (33). Previous studies have reported comparable diagnostic efficacy between corneal confocal microscopy and IENFD in diabetic neuropathy, providing additional support for the clinical value of corneal confocal microscopy as a surrogate marker for DPN (31, 34). Meta-analysis has provided robust evidence that corneal confocal microscopy can detect small nerve fibre loss in DPN (35). Our results showed that AHI was independently related to CNFL and CNFT, particularly CNFT. Using multivariate regression models, we found that AHI accounted for 17.1% of the total variance in CNFT. Among the four parameters of corneal confocal microscopy, CNFT shows the closest association with the severity of painful diabetic neuropathy and autonomic neuropathy, suggesting that CNFT serves as an effective morphological marker for SFN (36, 37). This indicates that OSA may play a significant role in SFN development in subjects with T2DM. CNFL also represents a valuable biomarker for DPN, with the advantage of strong measurement repeatability (38). Rapid reduction in CNFL may help identify subjects at highest risk of DPN development and progression (39). A lower level of CNFL indicates a higher future risk of DPN in T2DM subjects with OSA and the necessity of early intervention for these subjects. Meanwhile, CNFL may serve as a monitoring indicator for the therapeutic efficacy of continuous positive airway pressure intervention.

OSA, characterised by recurrent upper airway obstruction during sleep, induces chronic intermittent hypoxia which may contribute to the development or progression of DPN through several key mechanisms. Oxidative-nitrosative stress: Subjects with DPN exhibit higher serum levels of nitrotyrosine and lipid peroxides, indicating that oxidative-nitrosative stress contributes to DPN pathogenesis (40). This stress acts through mechanisms including reduced nerve perfusion, impaired vascular reactivity, and effects on various peripheral nervous system cells, and is linked to pain abnormalities and small sensory nerve degeneration. Inhibition of such stress improves experimental diabetic neuropathy (41). Nocturnal hypoxemia correlates with serum nitrotyrosine levels, suggesting this stress as a potential mechanistic link between OSA and DPN (15). Inflammation: Low-grade intraneural inflammation may represent a major facet of diabetic neuropathy (42). Intermittent hypoxia activates pro-inflammatory pathways, including Toll-like receptor (TLR)4/nuclear factor κ-light-chain-enhancer of activated B cells (NF-κB) and nucleotide-binding domain (NOD)-like receptor protein 3 (NLRP3) signalling pathways, leading to elevated levels of pro-inflammatory cytokines (such as IL-6 and IL-1β) (43). These pathways promote neuroinflammation, further compromising nerve integrity in diabetes (44, 45). Endothelial dysfunction: Endothelial impairment is sufficient to cause neuropathy (46). Endothelial dysfunction occurs early in the pathophysiology of diabetes and represents a strong, independent predictor of DPN, including small fibre neuropathy (47, 48). Meta-analysis has demonstrated that OSA, particularly moderate-severe OSA, appears to impair endothelial function, while continuous positive airway pressure treatment for OSA exerts positive effects on endothelial function (49, 50).

Although the relationship between OSA and DPN still requires further confirmation through rigorous randomised controlled trials (RCTs), research on their correlation may provide a new intervention direction for DPN prevention and treatment in T2DM subjects. Continuous positive airway pressure (CPAP) represents the standard treatment for OSA. Meta-analysis of RCTs indicates that CPAP therapy appears to significantly improve HbA1c in subjects with T2DM and OSA. These trials primarily focused on glycaemic control and had relatively short treatment durations (51). Comprehensive RCTs are required to evaluate the observed beneficial association between CPAP and DPN in T2DM subjects (52).

There are several limitations to our study. First, due to insufficient information, we were unable to conduct Neuropathy Disability Score (NDS) and Michigan Neuropathy Screening Instrument (MNSI) assessments to evaluate DPN severity. Second, while inflammatory factors contribute to DPN, their absence in our analysis represents an additional limitation. Third, the study lacked *a priori* sample size calculation, and the sample size was relatively small, with only 30 subjects having severe OSA, necessitating the combination of moderate and severe cases for analysis. Future studies with larger sample sizes are required to validate these findings. Fourth, as a cross-sectional study, it cannot establish causality; prospective cohort studies are needed to determine the relationship between OSA and DPN.

In summary, OSA was common among inpatients with T2DM. Subjects with T2DM and OSA showed higher odds of DPN. OSA was also correlated with small fibre damage in T2DM subjects. Further studies are required to determine whether OSA intervention can improve DPN in T2DM.

## Data Availability

The raw data supporting the conclusions of this article will be made available by the authors, without undue reservation.
