# Supplementary material for: The association between obstructive sleep apnoea and diabetic peripheral neuropathy in subjects with type 2 diabetes
Source: Front Endocrinol (Lausanne). 2025 Aug 29;16:1643826. doi: 10.3389/fendo.2025.1643826 (PMC12425751; doi:10.3389/fendo.2025.1643826)
Supplement: Supplementary file 1 [file DataSheet1.docx]

Supplementary material: Diagnostic process for diabetic peripheral neuropathy.

Signs

Clinical signs of neuropathy include numbness and pain.

Symptoms

Clinical Symptoms of neuropathy include ankle reflexes, vibration perception, light-touch perception (10-g monofilament), thermal (cold/hot) discrimination, and pinprick sensation.

Ankle reflexes

The subject with type 2 diabetes was placed in a supine position with the knee flexed at 90°. The examiner's left hand dorsiflexed the foot, and the right hand struck the Achilles tendon with a reflex hammer. Absence of ankle reflex was defined as no plantar flexion of the foot; diminished reflex was characterised by indistinct plantar flexion; and hyperactive reflex was noted when plantar flexion occurred upon slight touch. A positive result was determined when bilateral ankle reflexes were simultaneously diminished or absent.

Vibration perception

The stem of a 128-Hz tuning fork was placed on the dorsal bony prominence of the proximal phalanx of both great toes. With the subject's eyes closed, they were asked to report whether they could perceive the vibration. A positive result was defined as absence of vibration perception on either side.

Light-touch perception (10-g monofilament)

A 10-g monofilament nylon thread was applied to the dorsal aspect of both great toes with sufficient pressure to bend it for 1-2 seconds. The test was repeated four times per side, scoring one point for each unperceived stimulus. A positive result was defined as a total score of ≥5 points.

Thermal (cold/hot) discrimination

With the subject's eyes closed, the two ends of the testing device (a cold metal end and a warm polyester end) were applied to any point on the dorsal skin of the foot – specifically avoiding calluses, ulcers, scars, and necrotic tissue – for 1-2 seconds per stimulus. Temperature sensation abnormality was defined as the subject's inability to correctly identify the temperature difference between the two ends. A positive result was confirmed when this abnormality was present on either side.

Pinprick sensation

The dorsum of the foot was systematically pricked with a pin in a distal-to-proximal direction. Abnormal pain sensation was defined as either analgesia or hyperalgesia. A positive result was recorded when abnormal sensation was detected on either side.

Electromyography

Electromyographic testing was performed using a Nicolet Viking IV system (Nicolet Biomedical, USA) to measure latency, amplitude and conduction velocity in the posterior tibial, common peroneal, sural and superficial peroneal nerves of both lower limbs. All examinations were conducted by qualified medical personnel. The following reference values were considered abnormal:

The reference values of nerve conduction velocity.

| Nerve | Age (years) | Latency (ms) | Amplitude (mV) | Conduction velocity (m/s) |
| --- | --- | --- | --- | --- |
| Posterior tibial nerve | ≤50 | <6 | >7 | ≥37 |
|  | >50 | <6 | >4 | ≥37 |
| Common peroneal nerve | ≤50 | <5.5 | >2 | ≥37 |
|  | >50 | <5.5 | >2 | ≥37 |
| Sural nerve | ≤50 | NA | >4 | >40 |
|  | >50 | NA | >2 | >40 |
| Superficial peroneal nerve | ≤50 | NA | >6 | >40 |
|  | >50 | NA | >4 | >40 |

Abnormality criteria: Latency, amplitude or conduction velocity outside reference ranges; >50% amplitude reduction between ipsilateral proximal and distal common peroneal nerve sites; >50% inter-side amplitude difference; >10% inter-side conduction velocity reduction.

The diagnosis of diabetic peripheral neuropathy

Diabetic peripheral neuropathy was defined according to the Guideline for the Prevention and Treatment of Type 2 Diabetes Mellitus in China (2024 Edition). Subjects meeting any of the following criteria were diagnosed with diabetic peripheral neuropathy: (1) presence of ≥1 clinical sign and ≥1 symptom, (2) presence of ≥2 symptoms, or (3) abnormal nerve conduction[2].
